# Disparities in well-being outcomes among medical students: a comparative study between medical students with and without disability

**DOI:** 10.1186/s12909-025-06770-2

**Published:** 2025-02-07

**Authors:** Henry J. Seaborne, Lara Z. Chehab, Nikhil Rajapuram, Amanda Sammann

**Affiliations:** 1 The Better Lab, University of California San Francisco, San Francisco, CA USA; 2 College of Letters and Science, University of California Berkeley, Berkeley, CA USA; 3 Department of Surgery, University of California San Francisco, San Francisco, CA USA

**Keywords:** Medical student, Mental health, Disabilities, Well-being, Burnout, Depression, Distress, Logistic regression, Interventions, Intersectionality

## Abstract

**Background:**

The strenuous demands of medical education often lead to adverse mental health outcomes among students. Despite extensive research on medical student distress, the unique challenges faced by students with disabilities remain understudied. This study aims to investigate the well-being of medical students with and without disabilities, by comparing levels of distress, risk and protective factors, and modifiable variables’ impact on distress.

**Methods:**

From September 2019 to February 2020, we implemented an exploratory observational study to survey medical students across the US, US territories, and Caribbean. Participants completed the Medical Student Wellbeing Survey (MSWS), which was used to assess well-being and capture extensive demographic data on these students. We retrospectively sorted participant data into cohorts based on disability status, then compared them using rates of well-being outcomes, logistic regression, and interaction analyses. We conducted logistic regression analyses to identify significant predictors of severe distress, burnout, and depression.

**Results:**

Of the 3162 medical student participants, 277 identified as having a disability. Respondents with disabilities reported significantly higher rates of severe distress (65%) compared to their non-disabled peers (51.3%). Additionally, burnout and depression rates were higher among disabled students, with 80.41% experiencing burnout and 54.84% experiencing depression. Logistic regression revealed that certain identities, as well as high debt and tuition costs, were significant predictors of severe distress across cohorts. Uniquely, marital status emerged as a protective factor specifically for students with disabilities.

**Conclusion:**

Medical students with disabilities exhibit higher levels of severe distress, burnout, and depression than their non-disabled counterparts. Current interventions and modifiable factors are insufficient in lessening severe distress for these students. These findings highlight the necessity for tailored support strategies and structural interventions to improve the well-being of medical students with disabilities, especially for those with intersecting vulnerable identities.

**Trial registration:**

This study does not report the results of a health care intervention on human participants, so trial registration is not required.

**Supplementary Information:**

The online version contains supplementary material available at 10.1186/s12909-025-06770-2.

## Background

Medical education is known for its rigorous demands and stressors, often leading to adverse mental health outcomes among students [1, 2]. Multiple studies from 2006 to 2016 have estimated that 27.2% of medical students suffer from depression, 11.1% have suicidal ideations, ​​49.6% experience burnout, and 28.7% present with anxiety; however, only 15.7% of students seek psychiatric therapy and support [3–5]. Furthermore, quality-of-life surveying instruments show that medical students score lower on both social and psychological metrics compared to public peer controls [6]. These factors emphasize the need for medical schools to better identify and support medical students in distress and have driven extensive research into models of resilience against poor well-being [7, 8]. However, despite widespread recognition and research on the issue of medical student distress, a subgroup of medical students has remained relatively understudied in the context of well-being: those with disabilities.

The Center for Disease Control and Prevention (CDC) defines disability as “any condition of the body or mind (impairment) that makes it more difficult for the person with the condition to do certain activities (activity limitation) and interact with the world around them (participation restrictions)” [9, 10]. Compared to definitions that only consider physical disabilities, this broad definition encompasses far more individuals, acknowledging the wide range of needs and assistance required for various impairments. Additionally, given that larger proportions of the population suffer from chronic conditions and aging, the prevalence of disability has only increased significantly in the modern era [11]. The 2019 Behavioral Risk Factor Surveillance System (BRFSS), which assessed disability by hearing, vision, cognition or mental, mobility, self-care, and independent living, estimated the prevalence of disability in the US to be 26.8% of noninstitutionalized adults aged 18 years or older [12]. Furthermore, in a study covering 60 medical schools, the prevalence of disability among medical students increased by 69% from 2016 to 2019 [13]. Given the estimated national disability prevalence and the increasing number of reported medical students with disability each year, it is imperative to explore the unique challenges presented by disability and how they connect to student well-being [13, 14]. Researchers have demonstrated that medical students with disabilities, ranging from learning impairments to mobility restrictions, struggle with attendance, poor concentration, difficulties with written work, financial stability, and performance in anatomy and surgery [15]. Yet little research exists on effective mental health interventions for medical students with disabilities, comparative well-being to peers, and the underlying factors contributing to the well-being disparities between students with and without disabilities. This knowledge gap emphasizes the importance of comprehensive research to identify the unique challenges faced by medical students with disabilities and to better inform medical institutions’ well-being interventions.

Since the COVID-19 pandemic, many system-based interventions have been implemented to correct the root causes of distress and burnout for physicians and medical students [16]. These include mindfulness-based interventions, incentivized team-based physical activities, and encouraged social engagement, among others [17–19]. While these interventions show promise in alleviating medical student distress, they lack standardized use across medical schools and tailored implementation for a range of diverse student identities. Accounting for identity and tailoring interventions is especially important given the compounding effects of vulnerabilities [20–22]. For the identity of underrepresented minorities, wellness and resilience attached to feelings of representation were improved when URM medical students were paired with and mentored by URM medical residents [23]. For female-identifying students, there has yet to be research showing the efficacy of tailored wellness interventions. Yet studies focused on wellness disparities by gender have proposed many interventions, such as sharing household tasks more equitably with their partner or significant other, increasing female mentorship and leadership, and preventing internalized gender-based stereotypes in medicine [24–26]. However, other than perspectives from practitioners, medical students, and a few studies on burnout for disabled persons, there are no existing studies that examine comparative well-being or effective interventions to establish well-being equity [27, 28]. Of those that studied burnout for disabled students, the data and analyses failed to capture the wide range of identities and characteristics student bodies have, such as gender, debt, and tuition [29, 30].

In this study, we seek to address this gap in the literature by conducting a comparative analysis of well-being indicators between medical students with and without disabilities. By examining levels of distress, identifying risk and protective factors, and evaluating the effectiveness of existing support strategies, we aim to provide medical education professionals with insights that can inform policy changes and institutional interventions to better support these underrepresented medical students.

## Methods

This cross-sectional observational study aimed to evaluate the prevalence of distress and contributing risk or protective factors among medical students with and without disabilities across the nation. We gathered data from electronic surveys prospectively from September 2019 to February 2020 and conducted retrospective analysis to achieve this goal (See Additional File 1). Surveys used were in accordance with the Declarations of Helsinki and approved prior to administration by the University of California San Francisco institutional review board. After administration, we cleaned data to ensure only fully completed surveys entered into the dataset. We categorized the participants by their disability status into three cohorts: Medical Students with Disability (MSWD), Medical Students without Disability (MSWoD), and Unidentified Disability Status (UDS).

### Participants

We included medical students enrolled in accredited allopathic (MD) or osteopathic (DO) medical schools within the US and Caribbean. Participation was voluntary, confidential, and required completion of the entire survey. We distributed the surveys to medical student liaisons (MSLs) at each school, who we contacted through the Association of American Medical Colleges (AAMC) medical student representative listserv, and they were not incentivized to participate. Given this, participant response tracking was unfeasible to attain. Additionally, social media platforms like Twitter and Facebook made surveys available [14].

**Fig. 1:**
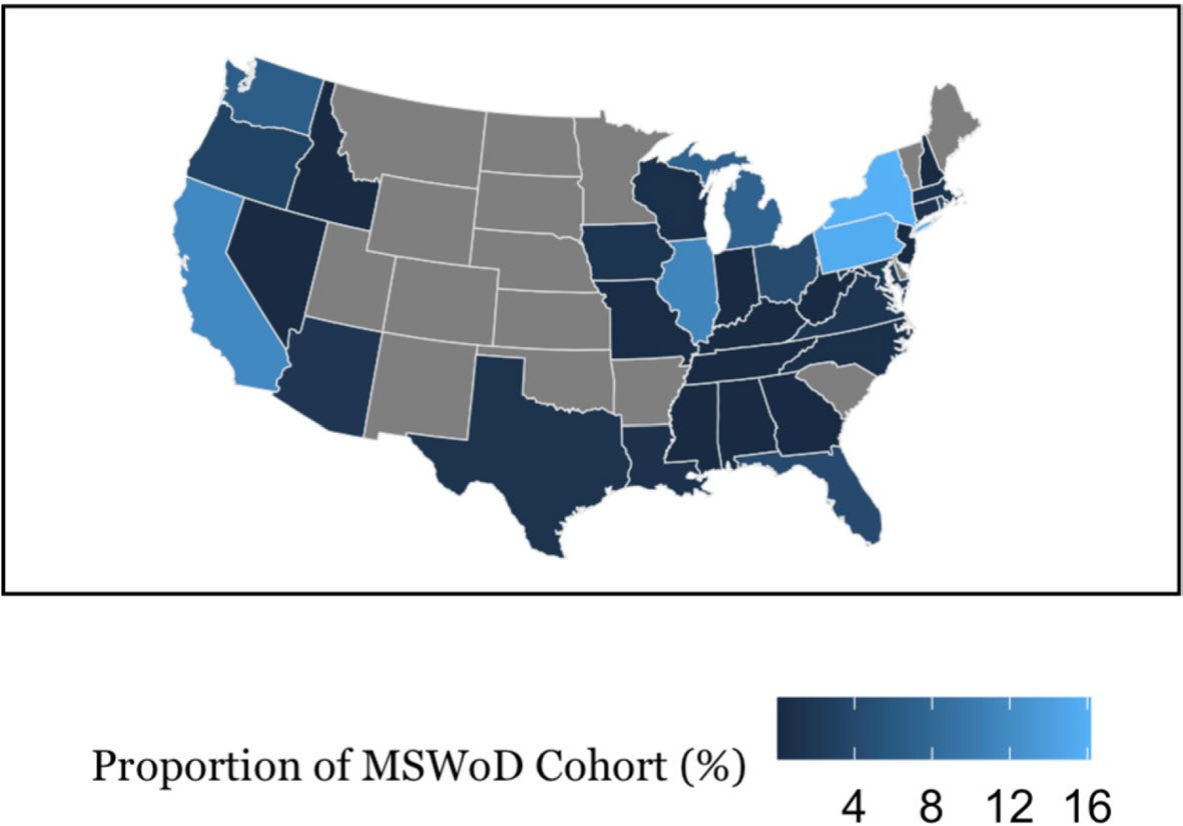
Rate of severe distress amongst MSWoD cohort by state Legend: Lighter color indicates higher rate of distress. Only states were included due to low counts of territories and Caribbean countries

**Fig. 2:**
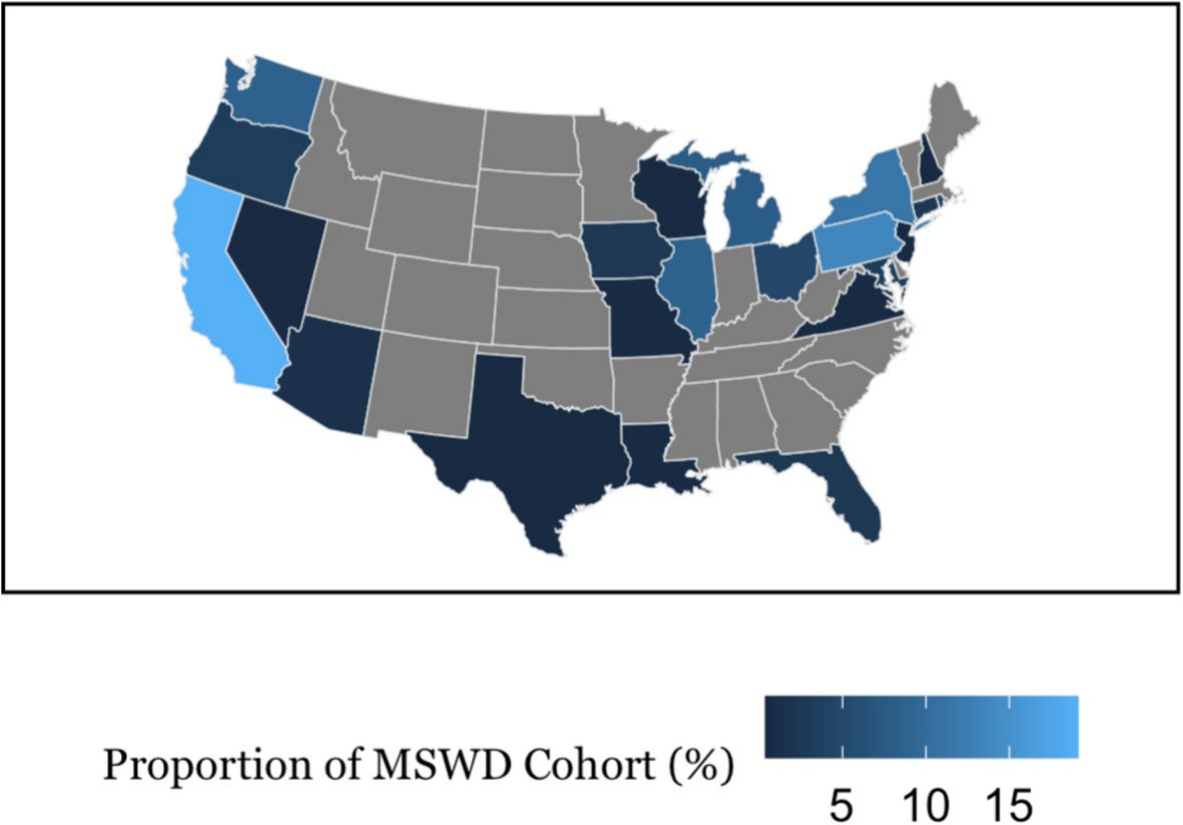
Rate of severe distress amongst MSWD cohort by state Legend: Lighter color indicates higher rate of distress. Only states were included due to low counts of territories and Caribbean countries

To gauge the spread of our cohorts across the nation, we mapped the distribution of participants who were assigned to both the MSWD or MSWoD cohorts (Figs. 1 & 2). Additionally to quantify distribution similarity we performed a cosine similarity test, which accounted for differences in sample sizes between MSWD and MSWoD cohorts. To perform this test, the ten states with the highest number of survey respondents for each cohort were selected and then used to calculate the cosine similarity test value.

To gauge counselor and resource use by participants, answers to MSWS survey questions “*At your institution, which of the following well-being resources are available? (Select all that apply)*” and “*At your institution, which of the following well-being resources have you utilized? (Select all that apply)*” were used (See Additional File 1). After data collection, participants were placed in either “Yes” or “No” for the variable Counselor Utilization. Participants were given different percentages, such as “20–40%”, for the variable Resource Utilization depending on the number of answer choices selected.

Survey Data Collection and Processing.

We used the MSWS to assess well-being, incorporating validated questions from the Medical Student Well-being Index (MS-WBI) and novel questions based on validated studies [31–34]. Medical student distress was assigned to individual participants given an MS-WBI score of N ≥ 4. Given the broad and imprecise definition of distress, we use it to broadly refer to depression, suicidal ideation, burnout, anxiety, and other related mental health issues [35]. The MS-WBI score of four or greater was the chosen threshold given sensitivity and specificity ≥ 90% for identifying students in severe distress with low mental quality of life, recent suicidal ideation, and serious thoughts of dropping out [36]. In addition to distress, we separately assigned medical student burnout and depression, based on their responses to validated questions within the MS-WBI [31, 37]. Besides well-being metrics, we also collected demographic data such as race and gender, taking into account their correlation with distress and the intersection of these identities and characteristics with disability. Potential modifiable variables used for interaction analysis included counselor utilization, resource utilization, tuition cost, specialty competitiveness, and debt burden. Before administering the national survey, 10 medical students from different medical institutions and demographics helped review and edit the MSWS survey for readability and representativeness in assessing medical student well-being.

The original survey was designed as part of a broader study aimed at examining general well-being outcomes among medical students. While it included a question to distinguish disability status, it was not explicitly developed for the purpose of studying disability. After collecting responses, we retrospectively categorized participants into three cohorts, MSWD, MSWoD, and UDS, based on their responses to the question, "*Do you currently identify as having a disability/chronic illness?*". Participants who answered "*Yes*" were placed in the MSWD cohort, "*No*" in the MSWoD cohort, and "*I’d Prefer Not to Say*" in the UDS cohort. Given the expansive list of disabilities that impact the structural and functional aspects of the body and mental functioning, participants were left to interpret whether they considered their own mental and or bodily state as disabled.

To address low participant numbers in certain categories, we performed variable recoding retrospectively when comparing the MSWD cohort against the MSWoD cohort. We compared the MSWD and MWSoD cohorts to the Combined cohort, which encompassed both disabled and non-disabled students. The variables recoded were Medical School Year, Marital Status, Debt, Tuition Average, Region, Specialty Competition, and Specialty Type, as explained.

For variable Medical School Year, MSWS participants selected schooling progress as either “*Pre-Clinical Coursework*” in the first and/or second year, “*Core Clerkships*” in the second and/or third year, “*Completed Core Clerkships*” in the third and/or fourth year, “*Gap Year*”, or “*Other*”. Variable choices “*Gap Year*” and “*Other*” were combined into “*Gap Year or Other*” to account for low counts in MSWD and MSWoD cohorts. For variable Marital Status, MSWS participants selected marital status as either “*Married*”, “*Never married*”, “*Widowed*”, or “*Divorced*”. Variable choices “*Never married*”, “*Widowed*”, and “*Divorced*” were combined into a new variable named “*Unmarried*” due to low counts in MSWD and MSWoD cohorts for those who identified as “*Widowed*” or “*Divorced*”. For variable Debt, MSWS participants selected either “ < *$20 K*”, “*$20—100 K*”, “*$100—300 K*”, or “ > *$300 K*”. Variable choices “*$20—100 K*”, *“$100—300 K*”, and “ > *$300 K*” were combined into “*X* > *$20 K*”, to distinguish students with substantial financial obligations from those with minimal debt. The *"X < $20K"* group likely includes a diverse population of students, such as those who benefited from family support, scholarships, or alternative funding sources that allowed them to minimize borrowing. While students with family resources or scholarships might be more common in this category, the grouping focuses on distinguishing levels of debt burden, as higher debt has a well-established association with financial stress and its impacts on well-being [38, 39]. For variable Tuition Average, MSWS participants selected either “*$0*”, “ < *$40 K*”, “*$40—$60 K*”, or “ > *$60 K*”. Variable choices “*$0*” and *“* < *$40 K*” as well as “*$40—$60 K*” and “ > *$60 K*” were combined so that participants were either categorized as having “X < *$40 K*” or “X > *$40 K*” for the cost of tuition yearly. Variables were combined to roughly distinguish the cost of in-state public colleges from out-of-state public colleges and both in-state and out-of-state private colleges [40]. For variable Region, MSWS participants identified their medical school, whose location was retrospectively categorized as either “*Northeast*”, “*West*”, or “*Non-coastal*” based on US Census Bureau data [41]. The variables “*Northeast*” and “*West*” were combined into a new variable, “*Coastal*”.

For variable Specialty Competition, MSWS participants selected their intended specialty choice and their confidence in the stated specialty option. In a previous study, specialty competitiveness was categorized based on 2018 National Resident Match Program data, with three categories of “*Low*”, “*Moderate*”, and “*High"* competition [14]. In this study, we combined “*Moderate*” and “*High*” competition to account for low MSWD cohort numbers. “*Moderate to High*” specialty competition included those interested in specialties with the United States Medical Licensing Examination (USMLE) Step 1 scores of > 230 or > 4% of unmatched applicants, respectively. For variable Specialty Type, MSWS participants were categorized into “*Medical*”, “*Surgical*”, or “*Mixed*” depending on their selected intended specialty. We combined the variable choices “*Surgical*” and “*Mixed*” into the variable name “*Surgical*”, given substantial time, energy, effort, and money to match into competitive residencies such as surgery [42, 43].

### Statistical analysis

Firstly, we conducted sub-analyses to estimate the rate of burnout and depression, as well as the risk of suicidal ideation, based on the distribution of distressed MS-WBI scores among students. We calculated burnout rates using MS-WBI questions 1 and 2, which asked "*Do you feel burned out from medical school?*" and "*Do you worry that medical school is hardening you emotionally?*" [36]. Depression rates were calculated based on MS-WBI question 3, “*During the past month*, *have you often been bothered by feeling down*, *depressed*, *or hopeless*?” [31]. Two-sample z-tests were conducted to confirm whether there are significant differences in the proportions of burnout and depression for our cohorts, MSWD and MSWoD. MS-WBI scores categorized as severely distressed ranged from a minimum of 4 to a maximum of 7. After creating the retrospective cohorts, we aggregated the number of MS-WBI scores greater than 4 and converted them into cohort-specific proportions to analyze the differences in the risk of suicidal ideation. To determine significant differences between the score proportions of the MSWD and MSWoD cohorts, two proportion z-tests were used. This approach allowed us to compare the distribution of distress (MS-WBI scores ≥ 4) between students with and without disabilities. Prior research has demonstrated a dose–response relationship between higher MS-WBI scores and a greater likelihood of suicidal ideation, with students in distress being at significantly higher risk compared to their peers without distress [44].

Second, univariate and multivariate logistic regression models were developed to investigate different risk and protective factors for severe distress, burnout, and depression among medical students with and without disability. These regression models produced crude and adjusted odd ratios and 95% confidence intervals for each factor, respectively. All statistical tests were two-sided and p-values of 0.05 or lower were considered statistically significant.

Finally, interaction analysis tested selected "modifiable" variables for synergistic or antagonistic effects on the relationship between disability status on severe distress. The variables tested included "Tuition Average", "Specialty Competitiveness", "Debt", "Coping with Counselor", and "Resource Utilization". We selected these variables because we categorized them as modifiable at the individual or institutional level. We performed all statistical analyses using R version 4.3.2.

## Results

### Demographics of disability cohorts

From the original MSWS dataset, 3162 medical student participants filled out the survey and 110 unique medical schools were represented. Out of these 3162 participants, we assigned approximately 2% (52) to the UDS cohort, 9% (277) to the MSWD cohort, and 89% (2758) to the MSWoD cohort. We tested statistical significance between the different cohorts using three-way analysis of variance tests (ANOVA) for continuous variables and Chi-square tests for categorical variables. Additionally, pairwise comparisons were made to prevent the UDS cohort from driving significance due to low counts. As shown in Table 1, for the pairwise analysis between MSWD and MSWoD cohorts "Medical School Year" (*p* = 0.017), "Gender" (*p* < 0.001), “Specialty Type” (*p* = 0.021), "Leave of Absence" (*p* < 0.001), and "Counselor Utilization" (*p* < 0.001) were the only demographic variables that were significantly.

**Table 1:** Demographic Characteristics of the Medical Student Cohorts

Variables	Variable Characteristics	Combined (%)	UDS (%)	MSWoD (%)	MSWD (%)	Overall *p*-value	MSWD vs. MSWoD *p*-value
n		3162	52	2758	277		
Medical School Progress (%)	Core Clerkships	641 (20.3)	18 (34.6)	558 (20.2)	49 (17.7)	0.008*	0.017*
	Gap Year or Oher	176 (5.6)	2 (3.8)	138 (5.0)	23 (8.3)		
	Completed Core Clerkships	709 (22.4)	8 (15.4)	635 (23.0)	49 (17.7)		
	Pre-Clinical	1636 (51.7)	24 (46.2)	1427 (51.7)	156 (56.3)		
Gender (%)	Male	1043 (33.8)	17 (32.7)	960 (34.8)	66 (23.8)	0.001*	
	Other	2043 (66.2)	35 (67.3)	1796 (65.2)	211 (76.2)		
Married (%)	Unmarried	2711 (87.9)	43 (82.7)	2428 (88.1)	239 (86.3)	0.343	0.421
	Married	374 (12.1)	9 (17.3)	327 (11.9)	38 (13.7)		
URM (%)	No	2667 (88.6)	40 (88.9)	2392 (88.9)	234 (86.3)	0.463	0.254
	Yes	342 (11.4)	5 (11.1)	300 (11.1)	37 (13.7)		
Debt (%)	*X* < 20 k	923 (31.8)	12 (28.6)	830 (31.9)	81 (30.8)	0.845	0.764
	*X* > 20 k	1984 (68.2)	30 (71.4)	1771 (68.1)	182 (69.2)		
Specialty Competitiveness (%)	Low	1464 (48.3)	23 (47.9)	1296 (47.9)	145 (52.7)	0.308	0.141
	Moderate to High	1567 (51.7)	25 (52.1)	1411 (52.1)	130 (47.3)		
Specialty Type (%)	Surgical	1450 (47.8)	21 (43.8)	1315 (48.6)	113 (41.1)	0.052	0.021*
	Medical	1581 (52.2)	27 (56.2)	1392 (51.4)	162 (58.9)		
Medical Program Type (%)	MD	3015 (95.8)	52 (100.0)	2625 (95.6)	269 (97.8)	0.064	0.106
	DO	131 (4.2)	0 (0.0)	122 (4.4)	6 (2.2)		
Medical Institution Type (%)	Public	1513 (48.1)	27 (51.9)	1314 (47.8)	144 (52.4)	0.312	0.171
	Private	1633 (51.9)	25 (48.1)	1433 (52.2)	131 (47.6)		
Region (%)	Coastal	2007 (63.5)	33 (63.5)	1735 (62.9)	184 (66.4)	0.511	0.275
	Non-Coastal	1155 (36.5)	19 (36.5)	1023 (37.1)	93 (33.6)		
City Characteristic (%)	Non-Metropolitan	1442 (45.9)	25 (48.1)	1246 (45.5)	133 (48.4)	0.617	0.391
	Metropolitan	1698 (54.1)	27 (51.9)	1495 (54.5)	142 (51.6)		
Tuition Average (%)	*X* < 40 k	427 (13.7)	2 (3.9)	385 (14.2)	35 (12.9)	0.096	0.611
	*X* > 40 k	2679 (86.3)	49 (96.1)	2327 (85.8)	237 (87.1)		
Leave of Absence (%)	Never Considered	2329 (78.7)	28 (57.1)	2149 (81.2)	151 (57.4)	< 0.001*	< 0.001*
	Considered	518 (17.5)	20 (40.8)	415 (15.7)	83 (31.6)		
	Have Taken	113 (3.8)	1 (2.0)	83 (3.1)	29 (11.0)		
Resource Utilization (%)	0—20%	975 (32.7)	13 (26.0)	895 (33.6)	64 (24.3)	< 0.001*	< 0.001*
	20—40%	696 (23.4)	13 (26.0)	630 (23.7)	53 (20.2)		
	40—60%	611 (20.5)	10 (20.0)	546 (20.5)	54 (20.5)		
	60—80%	397 (13.3)	5 (10.0)	345 (13.0)	47 (17.9)		
	80—100%	301 (10.1)	9 (18.0)	247 (9.3)	45 (17.1)		
Counselor Utilization (%)	No	2234 (75.1)	30 (60.0)	2042 (76.8)	159 (60.5)	< 0.001*	< 0.001*
	Yes	742 (24.9)	20 (40.0)	618 (23.2)	104 (39.5)		

Legend: Other includes Female, Transgender, Other, Prefer Not to Say

*URM* Underrepresented minority

^* ^Indicates a significance value of less than or equal to 0.05

Notably, the MSWD cohort had 11% more individuals identifying as “*Other*” (female, transgender, other, and prefer not to say), 8% more individuals having taken a leave of absence (LOA), 16% more individuals having considered a LOA, and 16% more individuals having used counselors compared to the MSWoD cohort.

The heatmap distribution of participants for the MSWD cohort (Fig. 1) and the MSWoD cohort (Fig. 2) are shown below. The cosine similarity test showed strong participant distribution similarity between cohorts given a value of 0.94, in a range from 0 to 1.

### Comparative estimation of well-being

The estimated rate of burnout, based on the first and second MS-WBI questions, was 80.41% for the MSWD cohort and 75.56% for the MSWoD cohort (*p* < 0.001). The estimated rate of depression, based on the third MS-WBI question, was 54.84% for the MSWD cohort and 47.93% for the MSWoD cohort (*p* < 0.001). The proportion of MS-WBI scores for students with disabilities was numerically higher at each scoring level compared to those without disabilities: 17.69% vs. 17.04% for a score of 4, 17.69% vs. 17.62% for a score of 5, 16.24% vs. 11.85% for a score of 6, and 13.35% vs. 4.79% for a score of 7. Following, two-proportion z-tests revealed significant differences between proportions of scores 6 (*p* = 0.04) and 7 (*p* < 0.001) for the MSWD and MSWoD cohorts.

### Univariate and multivariate logistic regression analysis for severe distress, burnout, and depression

Supplementary tables 2—10 present the results of the univariate and multivariate logistic regression analyses on severe distress, burnout, and depression for the cohorts Combined, MSWD, and MSWoD (See Additional Files 2—10). We organized the variables with statistical significance by theme into Identity, Financial, and Well-being, respectively.

### Identity regression results

In the Combined cohort, those identifying other than male (female, transgender, other) had higher odds of severe distress than males in both univariate (OR 1.45, 95% CI 1.25—1.65, *p* < 0.001) and multivariate models (OR 1.4, 95% CI 1.17—1.67, *p* < 0.001). In both MSWD and MSWoD cohorts, a gender identity other than male was associated with higher odds of experiencing severe distress in the univariate analysis. However importantly, when comparing the MSWD and MSWoD cohort odds ratios, of the students with identity other than male, those with disability had a higher odds ratio for severe distress (OR 1.79, 95% CI 1.02—3.15, *p* = 0.043 versus OR 1.41, 95% CI 1.20—1.65, *p* = 0.001). In the Combined and MSWoD cohorts, those identifying other than male had higher odds of burnout and depression in both univariate and multivariate models, yet significance is not seen for the MSWD cohort (See Additional Files 2—10).

In the MSWD cohort, being married was associated with reduced likelihood of severe distress when controlling for all other variables in this analysis (OR 0.38, 95% CI 0.15–0.95, *p* = 0.040). However, it was not a statistically significant protective factor when its effects were considered alone, and it was not a statistically significant protective factor in any of the regression models for the MSWoD or Combined cohorts (See Additional Files 2—4). Marital status did not significantly affect burnout or depression likelihood in univariate or multivariate regression models (See Additional Files 5—10).

In the Combined Cohort, URM students had increased severe distress odds in the univariate model (OR 1.36, 95% CI 1.09—1.72, *p* = 0.008), but not in the MSWD or MSWoD cohorts (See Additional Files 2—4). In the Combined cohort, URM medical students were more likely to be depressed in both models and in the MSWoD cohort, URM medical students were under only the univariate regression (See Additional Files 8 & 10).

### Financial regression results

In the Combined cohort, medical students with more than $20,000 in debt were more likely to experience severe distress in both univariate (OR 1.71, 95% CI 1.46—2.00, *p* < 0.001) and multivariate models (OR 1.63, 95% CI 1.37—1.97, *p* < 0.001). They were also more likely to experience burnout and depression in both models (See Additional Files 2, 5, 8). In the MSWD cohort, only the univariate model showed that students with more than $20,000 in debt were more likely to experience burnout, while debt was not a significant factor for severe distress or depression (See Additional Files 3, 6, 9).

In the Combined cohort, those with tuition costs over $40,000 per year were more likely to experience severe distress in both univariate (OR 1.44, 95% CI 1.18—1.78, *p* < 0.001) and multivariate models (OR 1.46, 95% CI 1.15—1.86, *p* = 0.002). They were also more likely to experience burnout and depression in both models (See Additional Files 2, 5, 8). In the MSWD cohort, higher tuition costs were not significantly associated with severe distress, burnout, or depression (See Additional Files 3, 6, 9).

### Well-being regression results

In the cohorts Combined, MSWD, and MSWoD, severe distress was more likely for students who had considered or taken an LOA compared to those who had not (See Additional Files 2—4). Those who had taken an LOA were less likely to be severely distressed (OR 3.68, 95% CI 2.42—5.7, *p* < 0.001 and OR 3.78, 95% CI 2.31- 6.41, *p* < 0.001) compared to those who had only considered it (OR 5.55, 95% CI 4.39—7.08, *p* < 0.001 and OR 5.28, 95% CI 4.08—6.91, *p* < 0.001). In the sub-analyses of burnout and depression, considering or taking an LOA was associated with higher odds of both outcomes for all three cohorts. Those who had taken an LOA had a lower likelihood of burnout and depression compared to those who had only considered it (See Additional Files 5—10).

In the Combined cohort, students with 20–40% resource utilization were less likely to be severely distressed compared to those with 0–20% use under the multivariate model (OR 0.77, 95% CI 0.61—0.96, *p* = 0.021). In the MSWD cohort, those with 80–100% resource utilization were more likely to be distressed compared to 0–20% use, under the multivariate model (OR 3.52, 95% CI 1.22—11.17, *p* = 0.024). In both the Combined and MSWoD cohorts, students who used counseling services (Counselor Utilization) were more likely to be distressed, burned out, and depressed under both models of regression. However, no significance was shown for the MSWD cohort for a higher likelihood of severe distress, burnout, or depression given Counselor Utilization against not (See Additional Files 2–10).

### Interaction results

The results of the analysis showed all interaction terms were not statistically significant to establish a synergistic or antagonistic effect for disability status on severe distress for the cohorts MSWD and MSWoD.

## Discussion

This study provides insights into the mental health challenges faced by medical students with disabilities, revealing a clear disparity in well-being compared to their non-disabled peers. Our data shows that while both medical students and individuals with disabilities are at high risk for poor mental health outcomes, those who belong to both groups face even greater difficulties. Specifically, the MSWD cohort is significantly more susceptible to severe distress and mental health issues, a finding that aligns with and extends existing research on the burnout of medical students and those with disabilities [29, 30]. This study goes beyond previous work by highlighting how intersecting identities, such as gender, further intensify these challenges.

Intersections between identities of vulnerability can exacerbate mental well-being outcomes, such as severe distress. For example, in the Combined cohort, gender identity emerged as a significant predictor, with females, transgender individuals, and those identifying as “other” exhibiting higher odds of severe distress, burnout, and depression, a pattern consistent with existing research [45, 46]. However, when divided into cohorts by disability status, those who have intersecting disability and gender identity other than male were at a higher risk of severe distress than those who are non-disabled and male identifying. Alone, gender minorities in medical education and healthcare often conceal their identities, face discriminatory comments and actions, and lack representation in the majority of surgical subspecialties and leadership roles [47, 48]. Due to this discrimination, it is shown that 87% of female medical students, 88% female residents, and 91% of female practicing surgeons have decreased job satisfaction, perception of self-efficacy, and colleague relationships [49]. Together, when minority identities of sex and disability status intersect, women with disabilities report significantly lower levels of self-esteem and mastery and higher levels of emotional reliance, which are associated with greater depressive symptoms [50, 51]. Other studies have demonstrated that the sexual orientation of medical students can also influence discrimination and mistreatment [52]. Going forward, the updated MSWS should investigate whether sexual orientation, such as gay, lesbian, or bisexual identities, intersects with disability to further increase the vulnerability to well-being. The intersecting identities of disability and URM were also expected to even further diminish well-being; however, no significant results were shown when students were separated by disability status [30, 53]. Of the few studies on the intersection of URM and disability, it has been shown that URM and Asian people facing co-occurring disability are at higher burnout risk than White peers without disability [30]. The next steps should distinguish between participants with single and co-occurring disabilities to assess any potential impact on well-being outcomes.

Additionally, marital status emerged as a protective factor against severe distress for MSWD, a finding not observed in the MSWoD cohort. Multiple studies have shown that being married or, more specifically, experiencing the supportive quality of a marital relationship can beneficially modulate stress, yet none have studied this specific impact on medical students with disabilities [54–56]. Based on the findings in this manuscript, supportive relationships, such as marital relationships, might be particularly beneficial for disabled students, necessitating further research into relationship dynamics as a buffer against distress and poor mental well-being.

Unsurprisingly, all cohorts of students who considered or took a LOA had a higher likelihood of severe distress, burnout, and depression, but those who took LOAs had lower rates of these issues compared to those who only considered it. This suggests that taking a LOA can positively impact a medical student’s mental health, as confirmed by prior studies [57]. Most importantly, both models showed that students with disabilities had higher odds ratios for severe distress, burnout, and depression, both for those considering a LOA and those who were already taking one, compared to the other cohorts. This underscores not only the disparities in wellbeing between individuals with and without disabilities, but also the necessity for modifications in the management and support of these school absences. Drawing from qualitative studies of those who have taken LOAs, medical students face stigma regarding career advancement and capability, little administrative support by medical institutions, and extra worry of how to support themselves away from their education [58]. These students, from dealing with the loss of loved ones to parental leave, require support as they transition from a paused education.

Wellness interventions in medical education and training seek to alleviate the widespread poor well-being outcomes of students and later physicians. However, most of these interventions, such as resiliency training, mindfulness practices, and self-care education, focus only on the individual level. While these interventions offer some benefits and are effective for certain individuals, they are often generalized and do not represent the specific needs of the diverse body of medical students, such as those with disabilities [59–61]. According to the interaction results, regardless of disability status, students' counselor utilization and resource use had no significant impact on protection from distress, burnout, or depression, thus calling for a deeper seeded change.

To effectively address the root causes of distress among medical students with disabilities (MSWD), it is essential for medical schools and educators to implement structural changes. These changes should go beyond individual coping mechanisms to include comprehensive support systems that directly address disproportionate well-being challenges faced by underrepresented minorities, identities, and especially students with disabilities. For example, student-led support programs could be restructured to pair first-year students with residents or senior medical students who share similar identities, such as sex, gender, disability type, or nationality. This “buddy system” has shown promise among URM groups and could be expanded to foster deeper, identity-specific support networks that offer a strong source of support and coping relief [23, 62]. Strengthening connections among disabled medical students, particularly in relation to their unique experiences during training and education, could help safeguard their well-being.

Medical students, including those with disabilities, have been shown in both our study and previous research to experience significant distress related to debt and the financial burdens of medical education [63]. One solution is moving towards endowment-based models such as that of the New York University (NYU) School of Medicine, where all matriculants have zero-cost tuition [64]. On top of relieving debt burden, a change like this can also consequently diversify the student body’s identities as personal finance barriers to education are removed. For disabled persons, this change may be especially enticing given the consistently higher cost of total health expenditures, out-of-pocket care, and burden of treatment [65, 66]. However, while NYU has seen a rise in general and underrepresented minority applicants (disability data undisclosed), critics argue that free tuition alone may not significantly enhance diversity and identities, as it can shift acceptances toward top-ranking individuals who may not need financial support [64, 67, 68]. This concern is particularly relevant for students with disabilities, who not only face the explicit costs of disability but also the structural barriers that may hinder their candidacy. To truly support these students, medical schools—particularly those with endowment-based systems—should adopt a more holistic admissions process. This process should value not only traditional metrics like Medical College Admission Test (MCAT) scores but also consider factors such as socioeconomic background, unequal opportunities, structural barriers, and the unique challenges faced by students with disabilities [69]. Such an approach would help alleviate the financial burden of medical school and better support those whose marginalization or identity makes them most in need.

Lastly, given that structural changes take time to design and implement, it is also crucial to better adapt individual-focused wellness interventions to support students with disabilities. Often the broader discourse on wellness overlooks accommodations for students with disabilities. The current wellness initiatives, which often focus on activities like mindfulness, exercise, and self-care, may inadvertently exclude students who face additional challenges in managing their health and academic responsibilities [70]. For these disabled students, wellness may not just be about engaging in self-care but about having access to necessary accommodations that enable full participation in their education. These accommodations should be seen as integral to wellness, ensuring that all learners, regardless of their mental and or physical abilities, are supported in their journey through medical training. To achieve this, medical institutions should involve and follow the lead of said students so that unique challenges of disability are properly and respectfully supported. In doing so, medical students with disabilities will help foster an equitable and supportive educational environment that is appropriate for them.

In addition to empowering medical students with disabilities to define their own wellness interventions, it is equally important to enhance the curriculum to teach all medical students how to effectively care for patients with disabilities [71]. Research highlights a gap in medical training regarding the treatment of patients with intellectual and developmental disabilities (IDD), leaving many students feeling ill-equipped to address the unique needs of this population [72]. To effectively close this gap, literature recommends a multimodal teaching approach, such as combined didactic lectures, panels with professionals, and clinical skills curricula. [71, 73]. Such training not only improves medical students’ knowledge and skills but also fosters empathy and a better understanding of the challenges faced by individuals with disabilities, potentially including their student peers. By addressing this educational deficit, medical institutions can ensure that future physicians are better prepared to provide equitable, compassionate care to all patients, thereby promoting inclusivity and reducing disparities within the healthcare system.

### Limitations and next steps

The significantly lower number of students who identify with a disability in our sample, compared to those who do not, impacts this study. The MSWD cohort's small sample size significantly reduced the statistical power of our analysis on risk factors and interaction modeling. Notably, 2% (63) of medical students intentionally chose not to respond to the MSWS's disability status question, which may be due to stigmatization towards self-identification with disability. Consequently, if true, leading to exclusion of potential data from the MSWD and MSWoD cohort comparisons. As previously mentioned, stigmatization around the self-identification and treatment of disabled persons is common among medical schools and practice. Yet medical education continues to grow more holistically, with some curriculums adapting special disability-focused material in partnership with disability advocates and academic disability specialists [74–76]. Given these changes and the age of our MSWS data set, we should re-administer the survey to gather data from current medical students. Another limitation of our study is potential confounding between variables Medical School Progress and Debt, as students who are further into their medical school progression will likely have higher amounts of debt compared to those just entering their education.

Additionally, we did not have data to stratify by type of disability. To allow for more granular analyses in the future, cross-sectional surveys focusing on medical student disability and its connection to severe distress should identify the specific disability the person is facing. Differentiating between cognitive, physical, and sensory disabilities will help develop distinctive interventions for better medical student well-being. Despite statistically insignificant interaction terms, this result showed that our study’s individual and institutional “modifiable” variables were unable to consistently modulate an effect between disability status on severe distress.

Future research should focus on expanding data collection and developing interventions to accommodate the diverse needs of all medical students, particularly those with disabilities. Given the limited sample size and the lack of differentiation by disability type, it is crucial to improve future data collection methods and ensure comprehensive representation to better understand and address the specific challenges faced by disabled medical students.

## Conclusion

This investigation into the well-being of medical students with and without disabilities reveals significant disparities in mental health outcomes, emphasizing the need for structural interventions that address more than just disability status and type. With the MSWD cohort notably experiencing higher rates of severe distress, burnout, depressive symptoms, and a higher risk of suicidal ideation, this suggests that the current medical education structure inadequately supports the needs of students with disabilities. These findings prompt medical institutions to consider identity-specific stressors and implement targeted support strategies to close the gap between mental health disparities among medical students.

## Supplementary Information

Additional file 1. “Medical Student Wellbeing Survey”, blank survey used to collect data for this study and previous others.

Additional file 2. “Severe Distress in the Combined Cohort”, data including odds ratios, confidence intervals, and significance concerning severe distress and the Combined Cohort.

Additional file 3. “Severe Distress in the MSWD Cohort”, data including odds ratios, confidence intervals, and significance concerning severe distress and the MSWD Cohort.

Additional file 4. “Severe Distress in the MSWoD Cohort”, data including odds ratios, confidence intervals, and significance concerning severe distress and the MSWoD Cohort.

Additional file 5. “Burnout in the Combined Cohort”, data including odds ratios, confidence intervals, and significance concerning burnout and the Combined Cohort.

Additional file 6. “Burnout in the MSWD Cohort”, data including odds ratios, confidence intervals, and significance concerning burnout and the MSWD Cohort.

Additional file 7. “Burnout in the MSWoD Cohort”, data including odds ratios, confidence intervals, and significance concerning burnout and the MSWoD Cohort.

Additional file 8. “Depression in the Combined Cohort”, data including odds ratios, confidence intervals, and significance concerning depression and the Combined Cohort.

Additional file 9. “Depression in the MSWD Cohort”, data including odds ratios, confidence intervals, and significance concerning depression and the MSWD Cohort.

Additional file 10. “Depression in the MSWoD Cohort”, data including odds ratios, confidence intervals, and significance concerning depression and the MSWoD Cohort.

## Data Availability

The dataset analyzed during this study are available in the figshare repository, https://doi.org/10.6084/m9.figshare.27122526.v1.

## Abbreviations

MSWS: Medical student wellness survey
CDC: Center for disease control and prevention
BRFSS: Behavioral risk factor surveillance system
URM: Underrepresented minority
MSWD: Medical students with disabilities
MSWoD: Medical students without disabilities
UDS: Unidentified disability status
MD: Doctor of medicine
DO: Doctor of osteopathy
MSLs: Medical student liaisons
AAMC: Association of American medical colleges
MS-WBI: Medical student well-being index
USMLE: United States medical licensing examination
ANOVA: Analysis of variance
LOA: Leave of absence
NYU: New York University
MCAT: Medical college admission test
IDD: Intellectual and developmental disabilities
