# Supplementary material for: Disparities in well-being outcomes among medical students: a comparative study between medical students with and without disability
Source: BMC Med Educ. 2025 Feb 7;25:199. doi: 10.1186/s12909-025-06770-2 (PMC11804037; doi:10.1186/s12909-025-06770-2)
Supplement: Supplementary file 1 — Additional file 1. “Medical Student Wellbeing Survey”, blank survey used to collect data for this study and previous others. [file 12909_2025_6770_MOESM1_ESM.pdf]

Table H: Depression in the MSWD Cohort

Variables	Variable Characteristics	Univariable Odds Ratio (95% CI)	P-value	Multivariable Odds Ratio (95% CI)	P-value
Medical School Progress (vs. Core Clerkships)	Gap Year or Other	1.33 (0.47 - 4.01)	$p = 0.601$	1.02 (0.27 - 4.07)	$p = 0.975$
	Completed Core Clerkships	0.92 (0.40 - 2.08)	$p = 0.835$	0.82 (0.29 - 2.30)	$p = 0.708$
	Pre-Clinical Coursework	0.71 (0.36 - 1.37)	$p = 0.317$	0.98 (0.42 - 2.25)	$p = 0.957$
Gender (vs. Male)	Other	1.48 (0.85 - 2.59)	$p = 0.167$	1.52 (0.74 - 3.16)	$p = 0.261$
Marital Status (vs. Unmarried)	Married	0.96 (0.48 - 1.94)	$p = 0.898$	0.50 (0.20 - 1.23)	$p = 0.132$
URM (vs. Not URM)	URM	1.83 (0.88 - 4.01)	$p = 0.116$	1.48 (0.58 - 3.92)	$p = 0.423$
Debt (vs. $X < 20$k)	$X > 20$k	1.31 (0.77 - 2.23)	$p = 0.311$	1.41 (0.74 - 2.72)	$p = 0.297$
Specialty Competitiveness (vs. Low)	Moderate to High	0.71 (0.44 - 1.16)	$p = 0.171$	1.22 (0.44 - 3.43)	$p = 0.706$
Specialty Type (vs. Surgical)	Medical	1.50 (0.92 - 2.45)	$p = 0.103$	2.36 (0.84 - 6.86)	$p = 0.107$
Medical Program Type (vs. MD)	DO	NA	NA	NA	$p = 0.988$
Medical Institution Type (vs. Public)	Private	0.78 (0.48 - 1.26)	$p = 0.306$	0.82 (0.43 - 1.54)	$p = 0.535$
Region (vs. Coastal)	Non-Coastal	1.89 (1.13 - 3.24)	$p = 0.017^*$	2.04 (1.05 - 4.07)	$p = 0.038^*$
City Characteristic (vs. Non-Metropolitan)	Metropolitan	0.80 (0.49 - 1.30)	$p = 0.371$	1.18 (0.64 - 2.19)	$p = 0.591$
Tuition Average (vs. $X < 40$k)	$X > 40$k	0.82 (0.39 - 1.69)	$p = 0.604$	1.46 (0.55 - 3.88)	$p = 0.447$
Leave of Absence (vs. Never Considered)	Considered	4.87 (2.66 - 9.29)	$p < 0.001^*$	5.41 (2.63 - 11.78)	$p < 0.001^*$
	Have Taken	3.29 (1.42 - 8.34)	$p = 0.008^*$	3.22 (1.15 - 9.84)	$p = 0.031^*$
Resource Utilization (vs. 0 - 20% use)	20 - 40%	0.49 (0.23 - 1.02)	$p = 0.060$	0.35 (0.14 - 0.86)	$p = 0.023^*$
	40 - 60%	0.93 (0.44 - 1.96)	$p = 0.853$	0.91 (0.36 - 2.31)	$p = 0.837$
	60 - 80%	0.79 (0.37 - 1.71)	$p = 0.553$	0.68 (0.26 - 1.77)	$p = 0.433$
	80 - 100%	1.98 (0.87 - 4.75)	$p = 0.113$	2.08 (0.75 - 6.07)	$p = 0.167$
Counselor Utilization (vs. No Counselor Utilization)	Counselor Utilization	1.60 (0.97 - 2.69)	$p = 0.070$	1.62 (0.83 - 3.19)	$p = 0.163$
