# Supplementary material for: Disparities in well-being outcomes among medical students: a comparative study between medical students with and without disability
Source: BMC Med Educ. 2025 Feb 7;25:199. doi: 10.1186/s12909-025-06770-2 (PMC11804037; doi:10.1186/s12909-025-06770-2)
Supplement: Supplementary file 2 — Additional file 2. “Severe Distress in the Combined Cohort”, data including odds ratios, confidence intervals, and significance concerning severe distress and the Combined Cohort. [file 12909_2025_6770_MOESM2_ESM.pdf]

Table I: Depression in the MSWoD Cohort

Variables	Variable Characteristics	Univariable Odds Ratio (95% CI)	P-value	Multivariable Odds Ratio (95% CI)	P-value
Medical School Progress (vs. Core Clerkships)	Gap Year or Other	1.00 (0.68 - 1.45)	$p = 0.980$	0.91 (0.57 - 1.44)	$p = 0.683$
	Completed Core Clerkships	0.60 (0.48 - 0.75)	$p < 0.001^*$	0.61 (0.47 - 0.80)	$p < 0.001^*$
	Pre-Clinical Coursework	0.68 (0.56 - 0.82)	$p < 0.001^*$	0.84 (0.67 - 1.06)	$p = 0.151$
Gender (vs. Male)	Other	1.29 (1.10 - 1.51)	$p = 0.002^*$	1.27 (1.05 - 1.53)	$p = 0.012^*$
Marital Status (vs. Unmarried)	Married	0.91 (0.72 - 1.15)	$p = 0.431$	0.92 (0.70 - 1.20)	$p = 0.535$
URM (vs. Not URM)	URM	1.35 (1.06 - 1.72)	$p = 0.014^*$	1.31 (0.99 - 1.73)	$p = 0.056$
Debt (vs. $X < 20$k)	$X > 20$k	1.60 (1.35 - 1.89)	$p < 0.001^*$	1.53 (1.26 - 1.85)	$p < 0.001^*$
Specialty Competitiveness (vs. Low)	Moderate to High	0.99 (0.85 - 1.16)	$p = 0.938$	1.03 (0.78 - 1.37)	$p = 0.823$
Specialty Type (vs. Surgical)	Medical	0.95 (0.82 - 1.11)	$p = 0.542$	0.96 (0.72 - 1.27)	$p = 0.771$
Medical Program Type (vs. MD)	DO	1.91 (1.32 - 2.81)	$p = 0.001^*$	2.19 (1.34 - 3.62)	$p = 0.002^*$
Medical Institution Type (vs. Public)	Private	0.95 (0.81 - 1.10)	$p = 0.467$	0.88 (0.73 - 1.07)	$p = 0.200$
Region (vs. Coastal)	Non-Coastal	1.08 (0.93 - 1.26)	$p = 0.319$	1.08 (0.89 - 1.31)	$p = 0.414$
City Characteristic (vs. Non-Metropolitan)	Metropolitan	1.02 (0.88 - 1.19)	$p = 0.794$	1.17 (0.97 - 1.41)	$p = 0.110$
Tuition Average (vs. $X < 40$k)	$X > 40$k	1.38 (1.11 - 1.72)	$p = 0.004^*$	1.38 (1.07 - 1.78)	$p = 0.014^*$
Leave of Absence (vs. Never Considered)	Considered	5.22 (4.09 - 6.72)	$p < 0.001^*$	4.81 (3.68 - 6.35)	$p < 0.001^*$
	Have Taken	3.77 (2.35 - 6.26)	$p < 0.001^*$	3.17 (1.85 - 5.64)	$p < 0.001^*$
Resource Utilization (vs. 0 - 20% use)	20 - 40%	1.03 (0.84 - 1.26)	$p = 0.782$	0.81 (0.64 - 1.02)	$p = 0.077$
	40 - 60%	1.01 (0.81 - 1.24)	$p = 0.962$	0.83 (0.65 - 1.06)	$p = 0.136$
	60 - 80%	1.06 (0.83 - 1.36)	$p = 0.639$	0.84 (0.63 - 1.13)	$p = 0.249$
	80 - 100%	2.17 (1.62 - 2.91)	$p < 0.001^*$	1.35 (0.96 - 1.89)	$p = 0.081$
Counselor Utilization (vs. No Counselor Utilization)	Counselor Utilization	2.04 (1.70 - 2.46)	$p < 0.001^*$	1.76 (1.42 - 2.18)	$p < 0.001^*$
