# Supplementary material for: Disparities in well-being outcomes among medical students: a comparative study between medical students with and without disability
Source: BMC Med Educ. 2025 Feb 7;25:199. doi: 10.1186/s12909-025-06770-2 (PMC11804037; doi:10.1186/s12909-025-06770-2)
Supplement: Supplementary file 3 — Additional file 3. “Severe Distress in the MSWD Cohort”, data including odds ratios, confidence intervals, and significance concerning severe distress and the MSWD Cohort. [file 12909_2025_6770_MOESM3_ESM.pdf]

Medical Student Well-being Survey

This is a national survey aimed at addressing the lack of data regarding the current state of medical student well-being. The survey is 5 pages and takes approximately 5-7 minutes to complete. All information from survey responses is completely confidential and will be analyzed in order to develop specific system-wide interventions to improve medical student well-being.

Section 1: Medical School & Stage

1.01 What medical school are you currently attending?

____

1.02 What stage in medical school are you currently in?

○ Pre-Clinical Coursework (usually 1st Year, occasionally 2nd Year or MD/PhD) (9)

○ Have begun participating in Core Clerkships (usually 3rd Year, occasionally 2nd Year or MD/PhD) (3)

○ Have completed Core Clerkships (usually 4th Year, occasionally 3rd Year or MD/PhD) (4)

○ Currently on a Gap Year (e.g. Research, MBA, MPH, etc.) (10)

○ Other: (6) ____

Section 2: Medical Student Well-Being Index

Over the last month, have you...
2.01 Felt burned out from medical school?

- ◯ Yes (1)
- ◯ No (2)

2.02 Worried that medical school is hardening you emotionally?

- ◯ Yes (1)
- ◯ No (2)

2.03 Often been bothered by feeling down, depressed, or hopeless?

- ◯ Yes (1)
- ◯ No (2)

2.04 Fallen asleep while sitting inactive in a public place?

- ◯ Yes (1)
- ◯ No (2)

2.05 Felt that all things you had to do were piling up so high that you could not overcome them?

- ◯ Yes (1)
- ◯ No (2)

2.06 Been bothered by emotional problems (such as feeling anxious, depressed, or irritable)?

- ◯ Yes (1)
- ◯ No (2)

2.07 Has your physical health interfered with your ability to do your daily work at home and/or away from home?

- Yes (1)
- No (2)

Section 3: Demographics

3.01 What is your current age?

- ○ Less than or equal to 21 (1)
- ○ 22 - 24 (5)
- ○ 25 - 27 (2)
- ○ 28 - 31 (3)
- ○ Greater than or equal to 32 (4)

3.02 How do you currently describe your gender identity?

- ○ Female (1)
- ○ Male (2)
- ○ Transgender (3)
- ○ Other (4)
- ○ I'd Prefer Not to Say (5)

3.03 How do you identify your race/ethnicity? (Select all that apply)

- ☐ Asian (1)
- ☐ Black/African (2)
- ☐ Caucasian (3)
- ☐ Hispanic/Latinx (4)
- ☐ Native American (5)
- ☐ Pacific Islander (6)
- ☐ Prefer Not to Answer (7)

☐ Other: (8) ____

3.04 Do you currently identify as having a disability/chronic illness?

○ Yes (1)

○ No (2)

○ I'd Prefer Not to Say (3)

3.05 What is your current marital status?

○ Never Married (1)

○ Separated (2)

○ Divorced (3)

○ Widowed (4)

○ Married (5)

3.06 How much financial debt do you currently have?

○ Between $20K and $100K (2)

○ Between $100K and $300K (3)

○ >$300K (4)

○ I'm Not Sure (5)

○ I'd Prefer Not to Say (6)

3.07 What is your intended specialty? (Note: If you have multiple or are unsure, please choose your current top choice)

- ◯ Anesthesiology (1)
- ◯ Dermatology (2)
- ◯ Emergency Medicine (3)
- ◯ Family Medicine (4)
- ◯ Internal Medicine (5)
- ◯ Internal Medicine/Pediatrics (6)
- ◯ Neurosurgery (8)
- ◯ Neurology (9)
- ◯ Obstetrics and Gynecology (10)
- ◯ Ophthalmology (27)
- ◯ Orthopaedic Surgery (11)
- ◯ Otolaryngology (12)
- ◯ Pathology (13)
- ◯ Pediatrics (14)
- ◯ Pediatric Neurology (30)
- ◯ Physical Medicine and Rehabilitation (15)
- ◯ Plastic Surgery (16)
- ◯ Psychiatry (17)
- ◯ Radiation Oncology (18)
- ◯ Radiology-Diagnostic (19)

○ Radiology-Interventional (29)

○ Surgery (20)

○ Thoracic Surgery (21)

○ Urology (26)

○ Vascular Surgery (23)

○ Other: (24) ____

3.08 How confident are you in your choice of intended specialty?

○ 100% (1)

○ 75% (2)

○ 50% (3)

○ 25% (4)

○ 0% (5)

Section 4: Stressors & Well-Being Experience

4.01 How often do you participate in the following activities in an ideal week vs a realistic week?

	Ideally			Realistically		
	≥ 5 days (1)	2-4 days (2)	≤ 1 day (3)	≥ 5 days (1)	2-4 days (2)	≤ 1 day (3)

Eat healthy (1)	○	○	○	○	○	○
Exercise (2)	○	○	○	○	○	○
Mindfulness practice (3)	○	○	○	○	○	○
Hobby (i.e. Read a book, Create art) (4)	○	○	○	○	○	○
Connect with a friend/loved one (5)	○	○	○	○	○	○

4.02 How do the following items affect your stress level?

	Significant Decrease in Stress (1)	Mild Decrease in Stress (2)	No Change in Stress or N/A (3)	Mild Increase in Stress (4)	Significant Increase in Stress (5)
Grades/Evaluations (12)	○	○	○	○	○
National Board Exams (13)	○	○	○	○	○
Isolation from friends/family (1)	○	○	○	○	○
Gender or Gender Identity (9)	○	○	○	○	○
Ethnicity or Ethnic Identity (10)	○	○	○	○	○
Financial debt (2)	○	○	○	○	○
Lack of control over schedule (7)	○	○	○	○	○
Specialty of choice (4)	○	○	○	○	○
Concern about workload in residency (5)	○	○	○	○	○
Uncertainty about future (8)	○	○	○	○	○
Disability/Chronic illness status (6)	○	○	○	○	○
Caregiving responsibilities (3)	○	○	○	○	○

4.03 Which of the following activities do you use to cope with difficult situations (or a difficult day on clinical rotation)? (Select all that apply)

- ☐ Talk to a friend/loved one (1)
- ☐ Consume alcohol or marijuana (2)
- ☐ Consume other drugs (7)
- ☐ Exercise (3)
- ☐ Watch TV/movie (4)
- ☐ Lie down (5)
- ☐ Listen to music (6)
- ☐ Mindfulness practice (8)
- ☐ Meet with a counselor (9)
- ☐ Other: (10) ____

4.04 How do you feel your well-being has changed in the following domains since beginning medical school?

	Significant Decrease (1)	Mild Decrease (2)	No Change (3)	Mild Increase (4)	Significant Increase (5)
PHYSICAL WELL-BEING (1)	○	○	○	○	○
SOCIAL WELL-BEING (2)	○	○	○	○	○
EMOTIONAL WELL-BEING (3)	○	○	○	○	○

4.05 Please mark the statement you most identify with:

○ I have considered taking a Leave of Absence from school for my personal well-being (1)

○ I have taken a Leave of Absence from school for my personal well-being (2)

○ I have never considered taking a Leave of Absence from school for my personal well-being (3)

Section 5: Medical School Characteristics

5.01 What is your school's grading system during **Pre-Clinical Courses**?

○ Pass/Fail ONLY (1)

○ Pass/Fail + Recognitions for Honors/High Pass (2)

○ Letter Grades (A, B, C, etc.) (3)

○ Other: (4) ____

5.02 How supportive do you feel your school's faculty are in terms of well-being?

○ Not Supportive at All (1)

○ Somewhat Supportive (2)

○ Strongly Supportive (3)

5.03 At your institution, which of the following well-being resources are available? (Select all that apply)

- ▢ Mental Health & Counseling Services (1)
- ▢ Peer Mentorship (2)
- ▢ Self-Care Education (4)
- ▢ Mindfulness/Meditation Classes (5)
- ▢ Community Building Events (3)
- ▢ Other: (6) ____

Carry Forward Selected Choices - Entered Text from "At your institution, which of the following well-being resources are available? (Select all that apply)"

5.04 At your institution, which of the following well-being resources have you utilized? (Select all that apply)

- ▢ Mental Health & Counseling Services (1)
- ▢ Peer Mentorship (2)
- ▢ Self-Care Education (3)
- ▢ Mindfulness/Meditation Classes (4)
- ▢ Community Building Events (5)
- ▢ Other: (6)

5.05 What well-being resource(s), if offered at your school, do you feel would be most useful?

____

5.06 If you have any further comments to share, please write them below:

____

Section 6: Interview Recruitment

6.01 Are you willing to be contacted for a short interview on your experience with medical student well-being?

- ◯ Yes (4)
- ◯ No (5)

Display This Question:

If Are you willing to be contacted for a short interview on your experience with medical student wel... = Yes

6.02 Please enter your school email address below:

____
