# Supplementary material for: Disparities in well-being outcomes among medical students: a comparative study between medical students with and without disability
Source: BMC Med Educ. 2025 Feb 7;25:199. doi: 10.1186/s12909-025-06770-2 (PMC11804037; doi:10.1186/s12909-025-06770-2)
Supplement: Supplementary file 4 — Additional file 4. “Severe Distress in the MSWoD Cohort”, data including odds ratios, confidence intervals, and significance concerning severe distress and the MSWoD Cohort. [file 12909_2025_6770_MOESM4_ESM.pdf]

Table A: Severe Distress in the Combined Cohort

Variables	Variable Characteristics	Univariable Odds Ratio (95% CI)	P-value	Multivariable Odds Ratio (95% CI)	P-value
Medical School Progress (vs. Core Clerkships)	Gap Year or Other	0.97 (0.68 - 1.38)	$p = 0.843$	0.92 (0.59 - 1.43)	$p = 0.697$
	Completed Core Clerkships	0.56 (0.45 - 0.69)	$p < 0.001^*$	0.52 (0.40 - 0.68)	$p < 0.001^*$
	Pre-Clinical Coursework	0.62 (0.51 - 0.74)	$p < 0.001^*$	0.77 (0.61 - 0.96)	$p = 0.018^*$
Gender (vs. Male)	Other	1.45 (1.25 - 1.68)	$p < 0.001^*$	1.40 (1.17 - 1.67)	$p < 0.001^*$
Marital Status (vs. Unmarried)	Married	1.03 (0.83 - 1.28)	$p = 0.812$	1.04 (0.81 - 1.35)	$p = 0.737$
URM (vs. Not URM)	URM	1.36 (1.09 - 1.72)	$p = 0.008^*$	1.26 (0.97 - 1.65)	$p = 0.088$
Debt (vs. $X < 20$k)	$X > 20$k	1.71 (1.46 - 2.00)	$p < 0.001^*$	1.63 (1.37 - 1.96)	$p < 0.001^*$
Specialty Competitiveness (vs. Low)	Moderate to High	1.12 (0.97 - 1.29)	$p = 0.113$	1.27 (0.97 - 1.67)	$p = 0.085$
Specialty Type (vs. Surgical)	Medical	0.92 (0.79 - 1.06)	$p = 0.226$	1.05 (0.80 - 1.38)	$p = 0.729$
Medical Program Type (vs. MD)	DO	2.18 (1.49 - 3.23)	$p < 0.001^*$	2.12 (1.26 - 3.55)	$p = 0.004^*$
Medical Institution Type (vs. Public)	Private	1.02 (0.88 - 1.17)	$p = 0.829$	1.00 (0.83 - 1.19)	$p = 0.959$
Region (vs. Coastal)	Non-Coastal	1.23 (1.07 - 1.43)	$p = 0.005^*$	1.27 (1.06 - 1.52)	$p = 0.011^*$
City Characteristic (vs. Non-Metropolitan)	Metropolitan	0.99 (0.86 - 1.15)	$p = 0.936$	1.13 (0.95 - 1.36)	$p = 0.169$
Tuition Average (vs. $X < 40$k)	$X > 40$k	1.44 (1.18 - 1.78)	$p < 0.001^*$	1.46 (1.15 - 1.86)	$p = 0.002^*$
Leave of Absence (vs. Never Considered)	Considered	5.55 (4.39 - 7.08)	$p < 0.001^*$	5.28 (4.08 - 6.91)	$p < 0.001^*$
	Have Taken	3.68 (2.42-5.79)	$p < 0.001^*$	3.78 (2.31 - 6.41)	$p < 0.001^*$
Resource Utilization (vs. 0 - 20% use)	20 - 40%	0.95 (0.78 - 1.16)	$p = 0.617$	0.77 (0.61 - 0.96)	$p = 0.021^*$
	40 - 60%	1.05 (0.86 - 1.29)	$p = 0.637$	0.88 (0.70 - 1.12)	$p = 0.302$
	60 - 80%	1.08 (0.86 - 1.37)	$p = 0.506$	0.88 (0.67 - 1.16)	$p = 0.367$
	80 - 100%	2.08 (1.59 - 2.74)	$p < 0.001^*$	1.34 (0.98 - 1.85)	$p = 0.070$
Counselor Utilization (vs. No Counselor Utilization)	Counselor Utilization	1.69 (1.43 - 2.00)	$p < 0.001^*$	1.38 (1.13 - 1.70)	$p = 0.002^*$
