# Supplementary material for: Disparities in well-being outcomes among medical students: a comparative study between medical students with and without disability
Source: BMC Med Educ. 2025 Feb 7;25:199. doi: 10.1186/s12909-025-06770-2 (PMC11804037; doi:10.1186/s12909-025-06770-2)
Supplement: Supplementary file 5 — Additional file 5. “Burnout in the Combined Cohort”, data including odds ratios, confidence intervals, and significance concerning burnout and the Combined Cohort. [file 12909_2025_6770_MOESM5_ESM.pdf]

Table B: Severe Distress in the MSWD Cohort

Variables	Variable Characteristics	Univariable Odds Ratio (95% CI)	P-value	Multivariable Odds Ratio (95% CI)	P-value
Medical School Progress (vs. Core Clerkships)	Gap Year or Other	0.83 (0.29 - 2.43)	$p = 0.724$	0.51 (0.13 - 2.02)	$p = 0.333$
	Completed Core Clerkships	0.76 (0.32 - 1.76)	$p = 0.522$	0.77 (0.26 - 2.21)	$p = 0.622$
	Pre-Clinical Coursework	0.79 (0.39 - 1.55)	$p = 0.498$	1.06 (0.44 - 2.50)	$p = 0.894$
Gender (vs. Male)	Other	1.79 (1.02 - 3.15)	$p = 0.043^{*}$	1.90 (0.92 - 3.97)	$p = 0.085$
Marital Status (vs. Unmarried)	Married	0.70 (0.35 - 1.44)	$p = 0.326$	0.38 (0.15 - 0.95)	$p = 0.040^{*}$
URM (vs. Not URM)	URM	1.54 (0.73 - 3.48)	$p = 0.273$	1.10 (0.42 - 3.03)	$p = 0.848$
Debt (vs. $X < 20$k)	$X > 20$k	1.26 (0.73 - 2.17)	$p = 0.404$	1.28 (0.66 - 2.48)	$p = 0.466$
Specialty Competiveness (vs. Low)	Moderate to High	1.02 (0.62 - 1.69)	$p = 0.923$	2.07 (0.74 - 6.39)	$p = 0.178$
Specialty Type (vs. Surgical)	Medical	1.18 (0.71 - 1.95)	$p = 0.512$	2.86 (1.01 - 8.99)	$p = 0.057$
Medical Program Type (vs. MD)	DO	2.73 (0.43 - 52.71)	$p = 0.363$	NA	NA
Medical Institution Type (vs. Public)	Private	0.81 (0.49 - 1.33)	$p = 0.408$	0.77 (0.40 - 1.48)	$p = 0.440$
Region (vs. Coastal)	Non-Coastal	1.29 (0.76 - 2.22)	$p = 0.342$	1.42 (0.72 - 2.86)	$p = 0.314$
City Characteristic (vs. Non-Metropolitan)	Metropolitan	0.91 (0.55 - 1.50)	$p = 0.718$	1.02 (0.55 - 1.91)	$p = 0.948$
Tuition Average (vs. $X < 40$k)	$X > 40$k	1.47 (0.71 - 3.02)	$p = 0.294$	2.28 (0.87 - 6.04)	$p = 0.094$
Leave of Absence (vs. Never Considered)	Considered	4.13 (2.22 - 8.09)	$p < 0.001^{*}$	6.21 (2.83 - 14.80)	$p < 0.001^{*}$
	Have Taken	3.49 (1.42 - 9.89)	$p = 0.010^{*}$	4.87 (1.64 - 17.02)	$p = 0.007^{*}$
Resource Utilization (vs. 0 - 20% use)	20 - 40%	0.83 (0.39 - 1.73)	$p = 0.612$	0.66 (0.27 - 1.60)	$p = 0.360$
	40 - 60%	1.49 (0.70 - 3.22)	$p = 0.305$	1.40 (0.55 - 3.63)	$p = 0.484$
	60 - 80%	1.21 (0.56 - 2.65)	$p = 0.634$	1.42 (0.54 - 3.83)	$p = 0.476$
	80 - 100%	2.74 (1.16 - 6.91)	$p = 0.026^{*}$	3.52 (1.22 - 11.17)	$p = 0.024^{*}$
Counselor Utilization (vs. No Counselor Utilization)	Counselor Utilization	1.30 (0.78 - 2.21)	$p = 0.320$	1.04 (0.52 - 2.08)	$p = 0.922$
