# Supplementary material for: Disparities in well-being outcomes among medical students: a comparative study between medical students with and without disability
Source: BMC Med Educ. 2025 Feb 7;25:199. doi: 10.1186/s12909-025-06770-2 (PMC11804037; doi:10.1186/s12909-025-06770-2)
Supplement: Supplementary file 6 — Additional file 6. “Burnout in the MSWD Cohort”, data including odds ratios, confidence intervals, and significance concerning burnout and the MSWD Cohort. [file 12909_2025_6770_MOESM6_ESM.pdf]

Table C: Severe Distress in the MSWoD Cohort

Variables	Variable Characteristics	Univariable Odds Ratio (95% CI)	P-value	Multivariable Odds Ratio (95% CI)	P-value
Medical School Progress (vs. Core Clerkships)	Gap Year or Other	1.04 (0.71 - 1.54)	$p = 0.923$	1.02 (0.64 - 1.65)	$p = 0.923$
	Completed Core Clerkships	0.57 (0.45 - 0.72)	$p < 0.001^{*}$	0.54 (0.41 - 0.70)	$p < 0.001^{*}$
	Pre-Clinical Coursework	0.62 (0.51 - 0.75)	$p = 0.024^{*}$	0.77 (0.61 - 0.97)	$p = 0.024^{*}$
Gender (vs. Male)	Other	1.41 (1.20 - 1.65)	$p = 0.001^{*}$	1.39 (1.15 - 1.67)	$p = 0.001^{*}$
Marital Status (vs. Unmarried)	Married	1.06 (0.84 - 1.34)	$p = 0.452$	1.11 (0.85 - 1.46)	$p = 0.452$
URM (vs. Not URM)	URM	1.35 (1.06 - 1.72)	$p = 0.134$	1.24 (0.94 - 1.64)	$p = 0.134$
Debt (vs. $X < 20$k)	$X > 20$k	1.77 (1.50 - 2.10)	$p < 0.001^{*}$	1.69 (1.40 - 2.04)	$p < 0.001^{*}$
Specialty Competiveness (vs. Low)	Moderate to High	1.14 (0.98 - 1.32)	$p = 0.144$	1.23 (0.93 - 1.64)	$p = 0.144$
Specialty Type (vs. Surgical)	Medical	0.88 (0.76 - 1.03)	$p = 0.924$	0.99 (0.74 - 1.31)	$p = 0.924$
Medical Program Type (vs. MD)	DO	2.25 (1.53 - 3.38)	$p = 0.005^{*}$	2.10 (1.26 - 3.55)	$p = 0.005^{*}$
Medical Institution Type (vs. Public)	Private	1.04 (0.90 - 1.21)	$p = 0.985$	1.00 (0.83 - 1.21)	$p = 0.985$
Region (vs. Coastal)	Non-Coastal	1.24 (1.06 - 1.44)	$p = 0.020^{*}$	1.25 (1.04 - 1.52)	$p = 0.020^{*}$
City Characteristic (vs. Non-Metropolitan)	Metropolitan	1.01 (0.87 - 1.17)	$p = 0.173$	1.14 (0.94 - 1.38)	$p = 0.173$
Tuition Average (vs. $X < 40$k)	$X > 40$k	1.45 (1.16 - 1.80)	$p = 0.008^{*}$	1.40 (1.09 - 1.81)	$p = 0.008^{*}$
Leave of Absence (vs. Never Considered)	Considered	5.50 (4.26 - 7.19)	$p < 0.001^{*}$	5.14 (3.88 - 6.88)	$p < 0.001^{*}$
	Have Taken	3.70 (2.28 - 6.25)	$p < 0.001^{*}$	3.80 (2.14 - 7.08)	$p < 0.001^{*}$
Resource Utilization (vs. 0 - 20% use)	20 - 40%	0.94 (0.77 - 1.16)	$p = 0.031^{*}$	0.77 (0.61 - 0.98)	$p = 0.031^{*}$
	40 - 60%	1.01 (0.82 - 1.25)	$p = 0.281$	0.87 (0.68 - 1.12)	$p = 0.281$
	60 - 80%	1.02 (0.80 - 1.31)	$p = 0.194$	0.82 (0.62 - 1.10)	$p = 0.194$
	80 - 100%	1.88 (1.41 - 2.53)	$p = 0.289$	1.20 (0.86 - 1.69)	$p = 0.289$
Counselor Utilization (vs. No Counselor Utilization)	Counselor Utilization	1.70 (1.41 - 2.04)	$p = 0.001^{*}$	1.43 (1.15 - 1.78)	$p = 0.001^{*}$
