# Supplementary material for: Disparities in well-being outcomes among medical students: a comparative study between medical students with and without disability
Source: BMC Med Educ. 2025 Feb 7;25:199. doi: 10.1186/s12909-025-06770-2 (PMC11804037; doi:10.1186/s12909-025-06770-2)
Supplement: Supplementary file 7 — Additional file 7. “Burnout in the MSWoD Cohort”, data including odds ratios, confidence intervals, and significance concerning burnout and the MSWoD Cohort. [file 12909_2025_6770_MOESM7_ESM.pdf]

Table D: Burnout in the Combined Cohort

Variables	Variable Characteristics	Univariable Odds Ratio (95% CI)	P-value	Multivariable Odds Ratio (95% CI)	P-value
Medical School Progress (vs. Core Clerkships)	Gap Year or Other	0.45 (0.30 - 0.68)	$p < 0.001^*$	0.42 (0.26 - 0.69)	$p = 0.001^*$
	Completed Core Clerkships	0.48 (0.36 - 0.64)	$p < 0.001^*$	0.47 (0.34 - 0.65)	$p < 0.001^*$
	Pre-Clinical Coursework	0.41 (0.32 - 0.53)	$p < 0.001^*$	0.48 (0.36 - 0.63)	$p < 0.001^*$
Gender (vs. Male)	Other	1.58 (1.33 - 1.87)	$p < 0.001^*$	1.44 (1.18 - 1.76)	$p < 0.001^*$
Marital Status (vs. Unmarried)	Married	1.02 (0.79 - 1.32)	$p = 0.874$	0.89 (0.67 - 1.19)	$p = 0.427$
URM (vs. Not URM)	URM	1.23 (0.94 - 1.63)	$p = 0.145$	1.18 (0.87 - 1.63)	$p = 0.295$
Debt (vs. $X < 20$k)	$X > 20$k	1.89 (1.58 - 2.25)	$p < 0.001^*$	1.78 (1.47 - 2.17)	$p < 0.001^*$
Specialty Competitiveness (vs. Low)	Moderate to High	0.86 (0.72 - 1.01)	$p = 0.071$	0.99 (0.73 - 1.34)	$p = 0.956$
Specialty Type (vs. Surgical)	Medical	1.14 (0.97 - 1.35)	$p = 0.122$	1.07 (0.79 - 1.44)	$p = 0.670$
Medical Program Type (vs. MD)	DO	1.63 (1.03 - 2.69)	$p = 0.045^*$	2.09 (1.13 - 4.14)	$p = 0.025^*$
Medical Institution Type (vs. Public)	Private	0.91 (0.77 - 1.07)	$p = 0.252$	0.93 (0.76 - 1.14)	$p = 0.481$
Region (vs. Coastal)	Non-Coastal	1.03 (0.87 - 1.23)	$p = 0.722$	0.89 (0.73 - 1.09)	$p = 0.264$
City Characteristic (vs. Non-Metropolitan)	Metropolitan	0.97 (0.82 - 1.14)	$p = 0.678$	1.03 (0.84 - 1.27)	$p = 0.774$
Tuition Average (vs. $X < 40$k)	$X > 40$k	1.45 (1.15 - 1.82)	$p = 0.001^*$	1.46 (1.13 - 1.89)	$p = 0.004^*$
Leave of Absence (vs. Never Considered)	Considered	5.41 (3.85 - 7.85)	$p < 0.001^*$	4.28 (2.99 - 6.31)	$p < 0.001^*$
	Have Taken	2.21 (1.35 - 3.87)	$p = 0.003^*$	1.76 (1.01 - 3.27)	$p = 0.058$
Resource Utilization (vs. 0 - 20% use)	20 - 40%	1.01 (0.81 - 1.26)	$p = 0.924$	0.83 (0.65 - 1.06)	$p = 0.143$
	40 - 60%	1.14 (0.90 - 1.44)	$p = 0.273$	0.95 (0.72 - 1.24)	$p = 0.687$
	60 - 80%	1.40 (1.06 - 1.86)	$p = 0.021^*$	1.06 (0.77 - 1.47)	$p = 0.723$
	80 - 100%	1.71 (1.24 - 2.41)	$p = 0.001^*$	1.05 (0.73 - 1.55)	$p = 0.783$
Counselor Utilization (vs. No Counselor Utilization)	Counselor Utilization	1.60 (1.30 - 1.98)	$p < 0.001^*$	1.32 (1.04 - 1.69)	$p = 0.024^*$
