# Supplementary material for: Disparities in well-being outcomes among medical students: a comparative study between medical students with and without disability
Source: BMC Med Educ. 2025 Feb 7;25:199. doi: 10.1186/s12909-025-06770-2 (PMC11804037; doi:10.1186/s12909-025-06770-2)
Supplement: Supplementary file 8 — Additional file 8. “Depression in the Combined Cohort”, data including odds ratios, confidence intervals, and significance concerning depression and the Combined Cohort. [file 12909_2025_6770_MOESM8_ESM.pdf]

Table E: Burnout in the MSWD Cohort

Variables	Variable Characteristics	Univariable Odds Ratio (95% CI)	P-value	Multivariable Odds Ratio (95% CI)	P-value
Medical School Progress (vs. Core Clerkships)	Gap Year or Other	0.76 (0.17 - 3.98)	$p = 0.721$	0.64 (0.10 - 4.65)	$p = 0.640$
	Completed Core Clerkships	0.35 (0.10 - 1.04)	$p = 0.069$	0.33 (0.07 - 1.24)	$p = 0.114$
	Pre-Clinical Coursework	0.39 (0.13 - 0.99)	$p = 0.067$	0.38 (0.10 - 1.13)	$p = 0.107$
Gender (vs. Male)	Other	1.26 (0.63 - 2.42)	$p = 0.503$	1.47 (0.62 - 3.37)	$p = 0.370$
Marital Status (vs. Unmarried)	Married	1.38 (0.58 - 3.81)	$p = 0.500$	1.06 (0.36 - 3.51)	$p = 0.925$
URM (vs. Not URM)	URM	1.70 (0.68 - 5.16)	$p = 0.298$	1.34 (0.40 - 5.38)	$p = 0.649$
Debt (vs. $X < 20$k)	$X > 20$k	1.97 (1.04 - 3.69)	$p = 0.035^*$	1.96 (0.90 - 4.27)	$p = 0.088$
Specialty Competitiveness (vs. Low)	Moderate to High	1.15 (0.63 - 2.11)	$p = 0.643$	3.44 (1.04 - 13.48)	$p = 0.055$
Specialty Type (vs. Surgical)	Medical	1.08 (0.59 - 1.97)	$p = 0.802$	2.69 (0.80 - 10.61)	$p = 0.127$
Medical Program Type (vs. MD)	DO	1.23 (0.19 - 23.77)	$p = 0.853$	NA	NA
Medical Institution Type (vs. Public)	Private	0.61 (0.33 - 1.11)	$p = 0.111$	0.53 (0.23 - 1.17)	$p = 0.120$
Region (vs. Coastal)	Non-Coastal	0.95 (0.51 - 1.79)	$p = 0.865$	0.62 (0.27 - 1.42)	$p = 0.254$
City Characteristic (vs. Non-Metropolitan)	Metropolitan	0.99 (0.54 - 1.80)	$p = 0.972$	1.47 (0.69 - 3.16)	$p = 0.313$
Tuition Average (vs. $X < 40$k)	$X > 40$k	1.01 (0.39 - 2.34)	$p = 0.981$	2.01 (0.61 - 6.24)	$p = 0.233$
Leave of Absence (vs. Never Considered)	Considered	6.62 (2.74 - 19.79)	$p < 0.001^*$	7.20 (2.55 - 24.98)	$p = 0.001^*$
	Have Taken	3.68 (1.22 - 15.98)	$p = 0.040^*$	3.47 (0.96 - 16.82)	$p = 0.080$
Resource Utilization (vs. 0 - 20% use)	20 - 40%	0.65 (0.29 - 1.44)	$p = 0.289$	0.39 (0.14 - 1.02)	$p = 0.058$
	40 - 60%	1.67 (0.68 - 4.30)	$p = 0.272$	1.34 (0.44 - 4.27)	$p = 0.611$
	60 - 80%	2.80 (1.00 - 9.15)	$p = 0.063$	2.77 (0.80 - 11.44)	$p = 0.125$
	80 - 100%	2.67 (0.95 - 8.73)	$p = 0.077$	3.47 (0.91 - 17.36)	$p = 0.089$
Counselor Utilization (vs. No Counselor Utilization)	Counselor Utilization	1.22 (0.66 - 2.31)	$p = 0.538$	0.97 (0.42 - 2.28)	$p = 0.940$
