# Supplementary material for: Disparities in well-being outcomes among medical students: a comparative study between medical students with and without disability
Source: BMC Med Educ. 2025 Feb 7;25:199. doi: 10.1186/s12909-025-06770-2 (PMC11804037; doi:10.1186/s12909-025-06770-2)
Supplement: Supplementary file 9 — Additional file 9. “Depression in the MSWD Cohort”, data including odds ratios, confidence intervals, and significance concerning depression and the MSWD Cohort. [file 12909_2025_6770_MOESM9_ESM.pdf]

Table F: Burnout in the MSWoD Cohort

Variables	Variable Characteristics	Univariable Odds Ratio (95% CI)	P-value	Multivariable Odds Ratio (95% CI)	P-value
Medical School Progress (vs. Core Clerkships)	Gap Year or Other	0.43 (0.28 - 0.67)	$p < 0.001^*$	0.41 (0.25 - 0.70)	$p = 0.001^*$
	Completed Core Clerkships	0.50 (0.37 - 0.67)	$p < 0.001^*$	0.49 (0.35 - 0.69)	$p < 0.001^*$
	Pre-Clinical Coursework	0.41 (0.32 - 0.54)	$p < 0.001^*$	0.49 (0.36 - 0.66)	$p < 0.001^*$
Gender (vs. Male)	Other	1.61 (1.35 - 1.92)	$p < 0.001^*$	1.45 (1.18 - 1.78)	$p < 0.001^*$
Marital Status (vs. Unmarried)	Married	1.00 (0.77 - 1.31)	$p = 0.987$	0.87 (0.64 - 1.18)	$p = 0.357$
URM (vs. Not URM)	URM	1.19 (0.90 - 1.60)	$p = 0.236$	1.14 (0.83 - 1.60)	$p = 0.425$
Debt (vs. $X < 20$k)	$X > 20$k	1.88 (1.56 - 2.26)	$p < 0.001^*$	1.75 (1.43 - 2.15)	$p < 0.001^*$
Specialty Competitiveness (vs. Low)	Moderate to High	0.83 (0.69 - 0.99)	$p = 0.036^*$	0.93 (0.68 - 1.28)	$p = 0.673$
Specialty Type (vs. Surgical)	Medical	1.15 (0.97 - 1.38)	$p = 0.109$	1.05 (0.77 - 1.43)	$p = 0.775$
Medical Program Type (vs. MD)	DO	1.68 (1.06 - 2.82)	$p = 0.037^*$	1.93 (1.03 - 3.85)	$p = 0.048^*$
Medical Institution Type (vs. Public)	Private	0.92 (0.78 - 1.10)	$p = 0.376$	0.96 (0.77 - 1.19)	$p = 0.697$
Region (vs. Coastal)	Non-Coastal	1.05 (0.88 - 1.26)	$p = 0.582$	0.90 (0.72 - 1.11)	$p = 0.313$
City Characteristic (vs. Non-Metropolitan)	Metropolitan	0.95 (0.80 - 1.14)	$p = 0.591$	0.99 (0.80 - 1.24)	$p = 0.958$
Tuition Average (vs. $X < 40$k)	$X > 40$k	1.49 (1.18 - 1.89)	$p = 0.001^*$	1.45 (1.11 - 1.89)	$p = 0.006^*$
Leave of Absence (vs. Never Considered)	Considered	5.03 (3.49 - 7.53)	$p < 0.001^*$	4.03 (2.74 - 6.15)	$p < 0.001^*$
	Have Taken	2.11 (1.20 - 4.02)	$p = 0.015^*$	1.73 (0.91 - 3.58)	$p = 0.117$
Resource Utilization (vs. 0 - 20% use)	20 - 40%	1.06 (0.84 - 1.33)	$p = 0.650$	0.88 (0.68 - 1.14)	$p = 0.342$
	40 - 60%	1.11 (0.87 - 1.43)	$p = 0.388$	0.94 (0.71 - 1.24)	$p = 0.638$
	60 - 80%	1.28 (0.96 - 1.73)	$p = 0.098$	0.96 (0.69 - 1.35)	$p = 0.810$
	80 - 100%	1.54 (1.09 - 2.21)	$p = 0.016^*$	0.90 (0.61 - 1.34)	$p = 0.596$
Counselor Utilization (vs. No Counselor Utilization)	Counselor Utilization	1.66 (1.33 - 2.09)	$p < 0.001^*$	1.43 (1.10 - 1.86)	$p = 0.007^*$
