# Supplementary material for: Disparities in well-being outcomes among medical students: a comparative study between medical students with and without disability
Source: BMC Med Educ. 2025 Feb 7;25:199. doi: 10.1186/s12909-025-06770-2 (PMC11804037; doi:10.1186/s12909-025-06770-2)
Supplement: Supplementary file 10 — Additional file 10. “Depression in the MSWoD Cohort”, data including odds ratios, confidence intervals, and significance concerning depression and the MSWoD Cohort. [file 12909_2025_6770_MOESM10_ESM.pdf]

Table G: Depression in the Combined Cohort

Variables	Variable Characteristics	Univariable Odds Ratio (95% CI)	P-value	Multivariable Odds Ratio (95% CI)	P-value
Medical School Progress (vs. Core Clerkships)	Gap Year or Other	1.02 (0.72 - 1.44)	$p = 0.915$	0.92 (0.59 - 1.41)	$p = 0.688$
	Completed Core Clerkships	0.60 (0.48 - 0.74)	$p < 0.001^*$	0.60 (0.46 - 0.78)	$p < 0.001^*$
	Pre-Clinical Coursework	0.67 (0.56 - 0.81)	$p < 0.001^*$	0.84 (0.68 - 1.05)	$p = 0.123$
Gender (vs. Male)	Other	1.30 (1.12 - 1.52)	$p = 0.001^*$	1.25 (1.05 - 1.50)	$p = 0.013^*$
Marital Status (vs. Unmarried)	Married	0.91 (0.73 - 1.12)	$p = 0.369$	0.87 (0.67 - 1.12)	$p = 0.275$
URM (vs. Not URM)	URM	1.39 (1.11 - 1.75)	$p = 0.004^*$	1.31 (1.01 - 1.71)	$p = 0.043^*$
Debt (vs. $X < 20$k)	$X > 20$k	1.59 (1.36 - 1.86)	$p < 0.001^*$	1.53 (1.28 - 1.83)	$p < 0.001^*$
Specialty Competitiveness (vs. Low)	Moderate to High	0.97 (0.84 - 1.12)	$p = 0.646$	1.04 (0.80 - 1.37)	$p = 0.754$
Specialty Type (vs. Surgical)	Medical	1.00 (0.87 - 1.16)	$p = 0.954$	1.01 (0.77 - 1.33)	$p = 0.921$
Medical Program Type (vs. MD)	DO	1.95 (1.35 - 2.84)	$p < 0.001^*$	2.20 (1.36 - 3.59)	$p = 0.002^*$
Medical Institution Type (vs. Public)	Private	0.94 (0.82 - 1.08)	$p = 0.403$	0.90 (0.75 - 1.07)	$p = 0.241$
Region (vs. Coastal)	Non-Coastal	1.11 (0.96 - 1.29)	$p = 0.153$	1.14 (0.95 - 1.36)	$p = 0.165$
City Characteristic (vs. Non-Metropolitan)	Metropolitan	1.00 (0.87 - 1.15)	$p = 0.998$	1.17 (0.98 - 1.40)	$p = 0.086$
Tuition Average (vs. $X < 40$k)	$X > 40$k	1.32 (1.08 - 1.63)	$p = 0.008^*$	1.37 (1.08 - 1.76)	$p = 0.011^*$
Leave of Absence (vs. Never Considered)	Considered	5.20 (4.17 - 6.54)	$p < 0.001^*$	4.79 (3.74 - 6.16)	$p < 0.001^*$
	Have Taken	3.75 (2.49 - 5.80)	$p < 0.001^*$	3.16 (1.98 - 5.17)	$p < 0.001^*$
Resource Utilization (vs. 0 - 20% use)	20 - 40%	1.00 (0.83 - 1.22)	$p = 0.971$	0.77 (0.62 - 0.97)	$p = 0.024^*$
	40 - 60%	1.02 (0.83 - 1.25)	$p = 0.843$	0.83 (0.66 - 1.05)	$p = 0.130$
	60 - 80%	1.09 (0.86 - 1.38)	$p = 0.469$	0.85 (0.65 - 1.12)	$p = 0.251$
	80 - 100%	2.28 (1.75 - 3.00)	$p < 0.001^*$	1.44 (1.06 - 1.98)	$p = 0.022^*$
Counselor Utilization (vs. No Counselor Utilization)	Counselor Utilization	2.03 (1.71 - 2.41)	$p < 0.001^*$	1.74 (1.42 - 2.13)	$p < 0.001^*$
